# Characterization of Rhizobia for the Improvement of Soybean Cultivation at Cold Conditions in Central Europe

**DOI:** 10.1264/jsme2.ME19124

**Published:** 2020-01-30

**Authors:** Kun Yuan, Moritz Reckling, Maria Daniela Artigas Ramirez, Salem Djedidi, Izumi Fukuhara, Takuji Ohyama, Tadashi Yokoyama, Sonoko Dorothea Bellingrath-Kimura, Mosab Halwani, Dilfuza Egamberdieva, Naoko Ohkama-Ohtsu

**Affiliations:** 1 Institute of Global Innovation Research (GIR), Tokyo University of Agriculture and Technology, 3–5–8, Saiwai-cho, Fuchu-shi, Tokyo, 183–8509 Japan; 2 Institute of Agriculture, Tokyo University of Agriculture and Technology, 3–5–8, Saiwai-cho, Fuchu-shi, Tokyo, 183–8509 Japan; 3 Leibniz Centre for Agricultural Landscape Research (ZALF), Müncheberg, 15374, Germany; 4 Department of Agricultural Chemistry, Tokyo University of Agriculture, Tokyo, 156–8502, Japan; 5 Key Laboratory of Biogeography and Bioresource in Arid Land, Xinjiang Institute of Ecology and Geography, CAS, Urumqi, People’s Republic of China

**Keywords:** Germany, *Glycine max*, nitrogen fixation, rhizobia, MLSA

## Abstract

In central Europe, soybean cultivation is gaining increasing importance to reduce protein imports from overseas and make cropping systems more sustainable. In the field, despite the inoculation of soybean with commercial rhizobia, its nodulation is low. In many parts of Europe, limited information is currently available on the genetic diversity of rhizobia and, thus, biological resources for selecting high nitrogen-fixing rhizobia are inadequate. These resources are urgently needed to improve soybean production in central Europe. The objective of the present study was to identify strains that have the potential to increase nitrogen fixation by and the yield of soybean in German soils. We isolated and characterized 77 soybean rhizobia from 18 different sampling sites. Based on a multilocus sequence analysis (MLSA), 71% of isolates were identified as *Bradyrhizobium* and 29% as *Rhizobium*. A comparative analysis of the *nodD* and *nifH* genes showed no significant differences, which indicated that the soybean rhizobia symbiotic genes in the present study belong to only one type. One isolate, GMF14 which was tolerant of a low temperature (4°C), exhibited higher nitrogen fixation in root nodules and a greater plant biomass than USDA 110 under cold conditions. These results strongly suggest that some indigenous rhizobia enhance biological nitrogen fixation and soybean yield due to their adaption to local conditions.

Rhizobia form a symbiotic association with leguminous plants, which results in the formation of an organ called a nodule on the root of the host plant. Inside the root nodule, rhizobia convert atmospheric nitrogen to ammonia, which may then be utilized for plant growth. Biological nitrogen fixation (BNF) may reduce chemical fertilization and its detrimental effects on soil properties and the environment (Zahran, 1999). Approximately 120 rhizobial species in 15 genera of *α-proteobacteria* and *β-proteobacteria* have been documented for diverse legumes (Andrews and Andrews, 2017). According to the literature, slow growers include *Bradyrhizobium japonicum*, *B. elkanii*, *B. yuanmingense*, and *B. liaoningense*, while fast growers comprise *Mesorhizobium tianshanense* and *Ensifer fredii*, which are symbionts of soybean (*Glycine max* [L.]) grown in different regions of the world (Jordan, 1982; Kuykendall *et al.*, 1992; Xu *et al.*, 1995; Chen *et al.*, 2000; Camacho *et al.*, 2002; Yang *et al.*, 2006; Man *et al.*, 2008; Yang and Zhou, 2008; Han *et al.*, 2009; Wang *et al.*, 2009; Andrews and Andrews, 2017).

Soybean is the most important legume in the world and its seeds contain 40% protein and 20% oil (Arslanoglu *et al.*, 2011). The northeastern region of China, the Korean peninsula, and Japan are the origin of soybean (Li *et al.*, 2010). China, USA, Brazil, and Argentina are the largest producers in the world. In recent years, the demand for soybean has increased in Europe due to increasing consumer demand for soy as a healthy alternative to meat as well as to reduce the import of genetically modified soybean products from USA and South America. However, soybean cultivation is still relatively new in central Europe (Zimmer *et al.*, 2016) even though areas of soybean cultivation increased two-fold between 2013 and 2018 (Krautgartner *et al.*, 2018). There is also large potential to grow soybean under the cooler and less favorable environments in northern parts of Europe (Singleton *et al.*, 1985). The rhizobia inoculation of soybean is a sustainable practice to induce atmospheric nitrogen fixation and subsequently improve crop productivity and soil fertility (Keyser and Li, 1992). Commercialized rhizobia have been utilized to inoculate soybeans in several countries across Europe (Zimmer *et al.*, 2016). Nevertheless, when grown for the first time under these conditions, soybean showed low nodule formation and a poor yield (Giller, 2001). Commercial inoculants containing *B. japonicum* are used in Germany; however, even after inoculation, soybean often forms a smaller number of nodules (Zimmer *et al.*, 2016) than that in Japan. These findings indicate that the rhizobia used as inoculants are not suitable for the local environmental conditions of Germany, and, thus, the development of biofertilizers using native rhizobia that are more appropriate for local conditions is needed. Different environmental factors affect legume and rhizobia symbiosis, such as temperature (Zhang and Smith, 1996; Zimmer *et al.*, 2016), pH (Lin *et al.*, 2012), salinity (Song *et al.*, 2017), the origin of cultivars (Muhammad *et al.*, 2012), and the amount of nitrogen in soil (Ray *et al.*, 2006). A low soil temperature also affects nodulation (Zhang and Smith, 1996; Zhang *et al.*, 1996). The optimal temperature for BNF ranges between 20 and 30°C (Bordeleau and Prévost, 1994). Therefore, soybean does not show good nodulation or nitrogen fixation when soil temperature is between 8 and 15°C (Zimmer *et al.*, 2016). Soybean cultivars that adapt to low temperatures have been successfully generated through breeding in central Europe; however, efficient symbionts have not yet been established (Zimmer *et al.*, 2016). Therefore, it is important to isolate German soybean-rhizobia that are highly efficient at fixing nitrogen and increasing biomass at low temperatures, which may reduce the use of chemical fertilizers as well as negative environmental impacts (Watson *et al.*, 2017).

An analysis of the genetic diversity of rhizobia is important for increasing rhizobial inoculation efficiency under various environmental conditions. There is currently no information on the genetic resources of native rhizobia in Germany.

The objectives of the present study were to investigate the genetic background of soybean-associated root nodule bacteria in some German soils and identify efficient isolates that adapt and increase soybean production under local conditions.

We isolated soybean rhizobia from soil samples derived from soybean-cultivated and non-cultivated locations in the northern part of Germany. The isolates obtained were subjected to a phenotypic characterization of their stress tolerance (temperature, salt, and pH) and symbiotic performance. To understand the genetic background of these isolates, we analyzed three housekeeping genes (16S rRNA, *recA*, and *atpD*) and two symbiotic genes (*nodD* and *nifH*). The symbiotic properties of some isolates were assessed under optimal temperature conditions and cold conditions.

## Materials and Methods

### Soil samples and collection sites

**Fig. 1.**
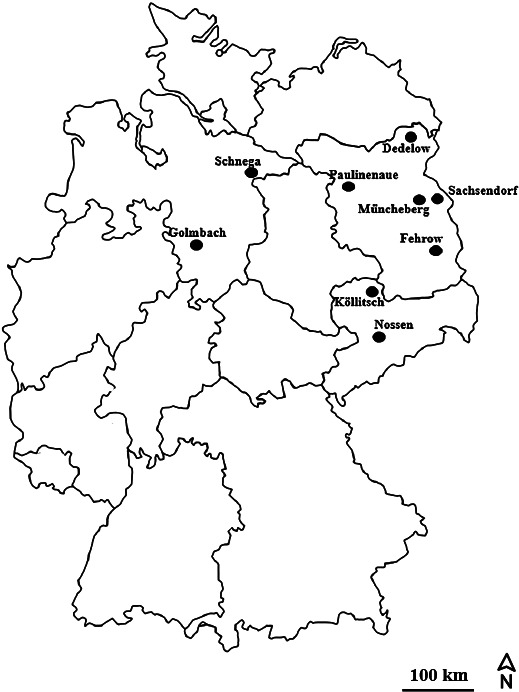
Map of Germany, indicating sampling sites. Soil samples were collected from 9 sites in the northern part of Germany, marked with black dots.

Eighteen soil samples were collected in November 2017 from 9 locations in the northern part of Germany (Fig. 1, Table 1). Samples were taken from different fields belonging to two different categories based on the presence (soil samples No. 10–18 in Table 1) or absence (No. 1–9) of a soybean cultivation history. At these locations, the pH of the corresponding soil samples ranged between 6.0 and 7.7, namely, slightly acidic to neutral. Prior to the isolation of rhizobia, all soil samples were transferred to the laboratory and kept at 4°C.

### Isolation of rhizobia from German soils

Two soybean cultivars “Merlin” and “Enrei” were used as trap hosts to isolate rhizobial strains. “Enrei” is a Japanese cultivar that is used worldwide under optimal cultivation. “Merlin” is a domesticated cultivar under German conditions and the most common cultivar for relatively cool growing conditions in Germany (Zimmer *et al.*, 2016). Soybean seeds were surface sterilized by immersion in 70% ethanol for 1‍ ‍min, and then in 3% sodium hypochlorite for 2‍ ‍min. Seeds were then thoroughly washed eight times with distilled water. Thereafter, surface-sterilized seeds were incubated at 28°C for 3‍ ‍d under dark conditions for germination. Germinated seeds were then transferred to 300-mL glass jars containing sterile vermiculite (Hirukon S, Hiruishi Kagaku Kogyo) watered with a sterilized nitrogen-free solution and irrigated to 60% of field capacity (Broughton and Dilworth, 1970). Inoculation was performed by the addition of 5 g of the soil sample diluted in 10 mL of sterilized water to each glass jar. Inoculated jars were transferred to a growth chamber and soybean plants were grown at 25°C for 4‍ ‍weeks under a 16-h light/8-h dark photoperiod. After 28‍ ‍d, prior to root nodule collection, entire soybean plants were removed from the glass jars and washed with tap water to remove vermiculite. Root nodules were detached and surface sterilized by immersion in 70% ethanol for 1‍ ‍min, followed by immersion in 3% sodium hypochlorite for 2‍ ‍min and rinsing five times with sterile water. Sterilized nodules were crushed in 0.5 mL of 15% glycerol and aliquots of the resultant suspensions were streaked on 1.5% yeast extract mannitol agar (YEM) plates (Somasegaran and Hoben, 1994). Plates were incubated at 28°C for 3–7 d. Single colonies were picked up, checked for purity by repeated streaking onto fresh YEM plates, and then used for further analyses.

### Abiotic stress tolerance test

The different phenotypic characteristics of the isolated bacteria, such as tolerance to low and high temperatures, NaCl levels, and low and high pH, were assessed as described previously (Djedidi *et al.*, 2011). The NaCl stress tolerance of isolates was tested on YEM medium containing 0, 1, 2, 3, and 4% (w/v) NaCl. Acid and alkaline stress tolerance was tested on YEM plates in which pH was adjusted to 4.5, 5, 6.8, 8, and 10 by the addition of HCl (1 M) or NaOH (1 M), respectively. The growth of rhizobia was examined after 7–14‍ ‍d of incubation at 28°C. The temperature tolerance of isolates was tested at 4, 15, 28, 37, and 45°C on YEM medium and bacterial growth was scored 7–14‍ ‍d after the inoculation (Djedidi *et al.*, 2011). These experiments were performed in three replications.

### Genomic DNA preparation

Isolates were grown in YEM broth at 28°C for 2–7 d. Rhizobial cells were collected using a centrifuge and then washed twice with sterile distilled water. Genomic DNA was extracted using a Wizard Genomic DNA purification kit (Promega).

### DNA amplification and sequencing

Polymerase chain reaction (PCR) amplification and sequencing of the DNA fragments of the 16S rRNA, *recA*, *atpD*, *nodD*, and *nifH* genes were performed. All genes were amplified by PCR using 10‍ ‍ng purified DNA, EX taq^TM^ (TaKaRa Bio). The pairs of primers utilized and amplification conditions are shown in Table S1. PCR products were purified using a FastGene Gel/PCR Extraction Kit (Nippon Genetics) and directly sequenced using the same primers used for PCR (Table S1). PCR products were sequenced using an ABI Prism 3500 Genetic Analyzer (Applied Biosystems), according to the manufacturer’s protocols.

### Phylogenetic analyses

Nucleotide sequences from the 16S rRNA, *recA*, *atpD*, *nodD*, and *nifH* genes of the isolates were used to evaluate their phylogenetic relationships. To identify the most closely related DNA sequences, they were compared to the GenBank nucleotide sequence database using BLAST. Multiple sequence alignment of the nucleotide sequences and phylogenetic trees were performed using the following software: Genetyx version 11, Clustal W, and MEGA version 7 (Tamura *et al.*, 2013). Phylogenetic trees were generated using the neighbor-joining method, the Kimura 2-parameter model. In the multilocus sequence analysis (MLSA) (Nzoué *et al.*, 2009), the phylogenetic tree was constructed using the 16S rRNA, *recA*, and *atpD* gene sequences obtained.

### Plant test

Rhizobial strains were tested for their nodulation ability and symbiotic performance on the soybean cultivars “Merlin” and “Enrei”. Seeds were surface-sterilized, as indicated in the isolation section. Seeds were allowed to germinate at 25°C for 3‍ ‍d in the dark. Seedlings were then transferred to glass jars containing sterile vermiculite and a nitrogen-free solution. A bacterial suspension containing approximately 10^7^ cells (2 mL plant^–1^) was used to inoculate each soybean plant. Plants were grown in a plant growth chamber at 25°C under a 16-h light/8-h dark photoperiod for 4‍ ‍weeks.

To imitate soybean growth conditions in Germany, another plant test was performed under low temperature conditions. Since the soybean growing season (between May and July, during which the average temperature is 17–24°C in daytime and 7–12°C in nighttime) in Germany has a 10°C difference between daytime and nighttime, plants were grown at 20°C under 16 h of light and 10°C under 8 h of dark for 40 d. Two soybean cultivars, “Merlin” and “Sultana”, were cultivated under low temperature conditions. These two cultivars are common for relatively cool growing conditions in Germany.

After harvesting the plants, shoot dry weight, root dry weight, root nodule dry weight, and nodule number were assessed. Plant shoots, roots, and nodules were dried at 80°C for 48 h to obtain dry weights. In the acetylene reduction assay (ARA), after harvesting the plants, roots that contained root nodules were placed in 300-‍mL glass jars, which had 10% of the air removed and replaced with 10% acetylene (v/v). Root nodules were then incubated at 25°C for 30‍ ‍min. The amount of ethylene was measured using a Shimadzu GC-2014 gas chromatograph (Shimadzu), equipped with a Porapak N column (Agilent Technologies).

### Nucleotide sequence accession numbers

DNA sequences were deposited in the DNA Data Bank of Japan (DDBJ) under the accession numbers LC434646 to LC434728 for 16S rRNA sequences, LC434729 to LC434811 for *recA* sequences, LC434812 to LC434894 for *atpD* sequences, LC434895 to LC434957 for *nodD* sequences, and LC434958 to LC435020 for *nifH* sequences.

## Results

### Isolation of rhizobia from German soils

A total of 454 indigenous isolates were obtained from the root nodules of the two soybean cultivars, with the following repartition: 264 isolates from “Merlin” and 190 strains from “Enrei”. Plants inoculated with soil samples with no soybean cultivation history, such as soil sample no. 1 (Sachsendorf), 2 (Dedelow), 3 (Paulinenaue), 4 (Nossen), 6 (Golmbach), and 7 (Schnega), did not show root nodule formation. Additionally, inoculations with soil samples 5 (Köllitsch), 8 (Müncheberg), and 9 (Müncheberg) resulted in only a few nodules (Table S2). On the other hand, we observed nodules and isolated rhizobia from soils with a soybean cultivation history, such as soils from the Müncheberg (Broughton and Dilworth, 1970; Chen *et al.*, 1991; Chen *et al.*, 2000; Camacho *et al.*, 2002) and Fehrow areas (Eckhardt *et al.*, 1931; Chen *et al.*, 1988; Frank *et al.*, 2008; Djedidi *et al.*, 2011; Chibeba *et al.*, 2017), using the two soybean cultivars “Merlin” and “Enrei” (Table S2). All the isolates produced similar creamy colonies, with a transparent light-yellow color when grown on YEM medium at 28°C. Regarding their growth properties, 85.2% of isolates were slow glowing (5–7 d) and the remaining 14.8% had fast growing (1–3 d) characteristics (Table S2).

### Abiotic stress tolerance of soybean rhizobia

**Fig. 2.**
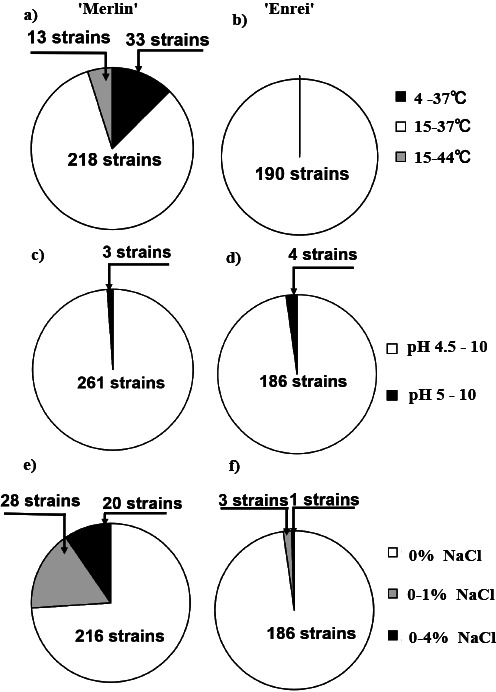
Abiotic stress tolerance of soybean rhizobia isolated from two soybean cultivars. (A, C, and E) isolated from “Merlin”; (B, D, and F) isolated from “Enrei”.

The responses of the 454 isolates to the different levels of temperature, salinity, and pH varied. Extreme (low/high) temperature, high salinity, and acidic/alkaline pH tolerant strains were identified among the screened soybean rhizobia (Fig. 2). Their phenotypic characteristics were investigated in more detail. The majority of the strains were able to grow at a temperature range of 15–37°C (Fig. 2). However, only 33 strains grew at 4°C and 13 strains were able to grow at a high temperature of 44°C. These low and high temperature tolerant strains were isolated from the “Merlin” cultivar. None of the strains isolated from “Enrei” soybeans grew at the low (4°C) or high temperature (44°C). A large number, corresponding to 98.5%, of strains were able to grow in a wide pH range, varying between 4.5 and 10. Additionally, only 31 strains were able to grow on medium amended with 1% NaCl; 28 were isolated from “Merlin” and 3 from “Enrei”. Twenty-one strains grew at salinity levels ranging between 1 and 4% NaCl; 20 were isolated from the “Merlin” cultivar and only 1 from “Enrei” (Fig. 2).

### Phylogenetic analysis of MLSA

**Fig. 3.**
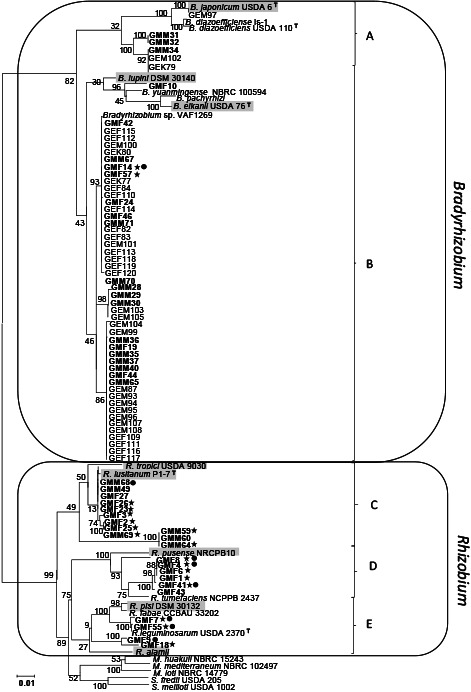
Phylogenetic tree of MLSA based on concatenated sequences of 16S rRNA (1,299 bp), *recA* (541 bp), and *atpD* (453 bp) genes. The nucleotide sequences from 77 representative isolates and reference strains of each species belonging to different genera. The tree was constructed by the neighbor-joining method. Bootstrap values are shown as percentages from 1,000 replicates. Scale bars represent 1% of the nucleotide substitutions. Strains isolated from “Merlin” were marked with a bold letter. Strains that survived at 4°C were marked with stars and strains that grew at 4% NaCl were marked with black dots.

Based on MLSA, 77 isolates were divided into two groups. The majority of the isolates were assigned to *Bradyrhizobium* (71.4%), and the remaining to *Rhizobium* (28.6%) (Fig. 3). In the *Bradyrhizobium* group, two subgroups were observed: subgroup A, containing 6 isolates from this study with reference stains* B. japonicum* USDA 6 and *B. diazoefficiens* USDA 110; subgroup B, containing 49 isolates with reference stains *B. lupini*, *B. yuanmingense*, *B. pachyrhizi*, *B. elkanii*, and *Bradyrhizobium* sp. VAF1269. Twenty-two isolates belonged to the *Rhizobium* cluster, which was also separated into three subgroups: subgroup C, containing 12 strains with the reference strains *R. tropici* USDA 9030 and *R. lusitanum* P1-7; subgroup D, containing 6 strains with the reference strain *R. pusense* NRCPB 10; and subgroup E, containing 4 strains with the reference strain *R. alamii*.

### Phylogenetic analysis of symbiotic genes for *nodD* and *nifH*

**Fig. 4.**
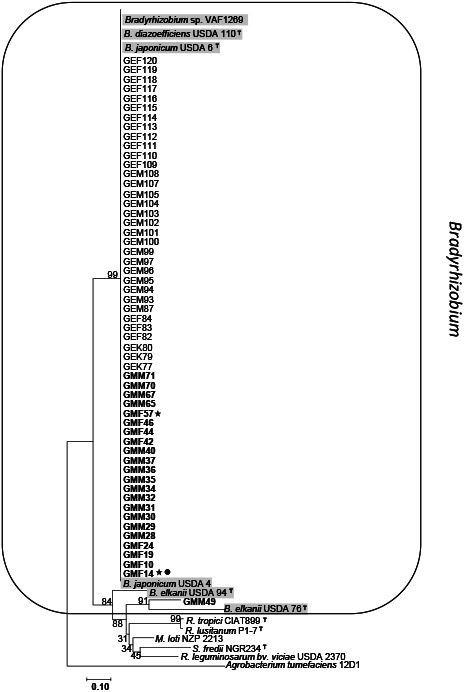
Phylogenetic tree of the *nodD* gene constructed using a 433-bp partial nucleotide sequence from 56 representative isolates and reference strains of each species belonging to different genera. Bootstrap values are shown as percentages from 1,000 replicates. Strains that survived at 4°C were marked with stars and strains that grew at 4% NaCl were marked with black dots.

**Fig. 5.**
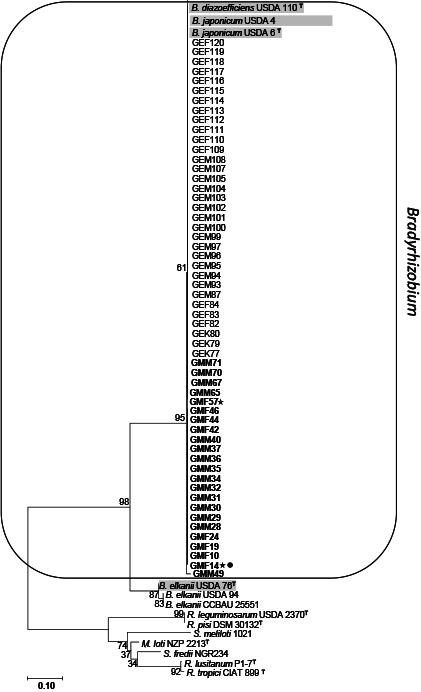
Phylogenetic tree of the *nifH* gene constructed using a 666-bp partial nucleotide sequence from 56 isolates and reference strains of each species belonging to different genera. Bootstrap values are shown as percentages from 1,000 replicates. Strains that survived at 4°C were marked with stars and strains that grew at 4% NaCl were marked with black dots.

We were unable to amplify the *nodD* and *nifH* genes in 21 out of 22 *Rhizobium* isolates (based on MLSA). Therefore, these isolates were not included in the phylogenetic trees in Fig. 4 for *nodD* and Fig. 5 for *nifH*. The phylogenetic tree, built with the partial sequences of the *nodD* genes for 56 isolates, showed two groups (Fig. 4). The first group of *Bradyrhizobium* was composed of 55 isolates and the reference strains of *Bradyrhizobium* sp. VAF1269, USDA 6, and USDA 4. The second group consisted of only one *Rhizobium* isolate, GMM49, which had a *nodD* gene that was very similar to that of *B. elkanii*. The phylogenetic analysis of the *nifH* genes showed similar results (Fig. 5). All 56 isolates, except for GMM49, had identical *nifH* genes, forming a large cluster together with *B. diazoefficiens* strains. A *nifH* gene of GMM49 showed lower (98%) similarity to that of *B. diazoefficiens*.

### Symbiotic performance under optimal temperature conditions

To elucidate the symbiotic properties of isolates, 44 representative strains; 22 belonging to *Bradyrhizobium* and 22 to *Rhizobium*, were selected based on MLSA phylogeny and inoculated into “Merlin” and “Enrei” (Fig. 3, Table 2). All plants inoculated with *Bradyrhizobium* isolates formed nodules (Table 2). Strains GMM71 and GMF14 induced a high biomass in both soybean cultivars. Regarding the “Merlin”’ cultivar inoculated with *Bradyrhizobium* isolates, the root nodule number ranged between 18.2±9.7 and 61.3±26.0 and ARA between 38.3±12.6 and 140.6±11.6‍ ‍μmol C_2_H_4_^–1^‍ ‍h^–1^‍ ‍g^–1^ dry weight of nodules (Table 2). Concerning “Enrei”, the root nodule number ranged between 24.3±10.7 and 48.7±16.2 and ARA between 11.1±7.2 and 94.8±7.3 μmol C_2_H_4_^–1^ h^–1^ g^–1^ dry weight of nodules (Table 2). However, no significant differences were observed in root nodule numbers or ARA between the two soybean cultivars.

Strain GMF14, isolated from soil sample 18, showed the highest root nodule number (61.3±26.0) when inoculated into “Merlin”. The plant biomass induced by this isolate was significantly higher than that of control plants for both soybean cultivars. The highest ARA for “Merlin” was obtained with isolate GMF24 (subgroup B) with 140.6±11.6 μmol C_2_H_4_^–1^ h^–1^ g^–1^ per dry weight of nodules. Isolates GEM96, GMM71, GMM36, GMM 34, GEM108, GMF 42, and GMF14 were the most effective in terms of plant growth promotion because they increased the soybean biomass by almost 2-fold from that of the non-inoculated control.

In the 22 isolates belonging to the *Rhizobium* group, only one isolate, GMM49, which was closely related to *R. tropici* and *R. lusitanum*, induced nodule formation on “Merlin” soybeans (Table 2). Regarding the “Merlin” cultivar inoculated with strain GMM49, the root nodule number was 35.7±13.2 and ARA was 119±28.6 μmol C_2_H_4_^–1^ h^–1^ g^–1^ dry weight of nodules.

### Symbiotic performance under low temperature conditions

To assess the abilities of some isolates for nodulation and nitrogen fixation on soybean plants under cold conditions, a second plant test was performed in which inoculated plants were maintained at low temperatures. Temperatures were set at 20°C in the daytime and 10°C at night, which corresponded to the average temperatures in Germany during the soybean sowing season (May to June). In this test, two German soybean cultivars “Merlin” and “Sultana”, known to be adapted to cold conditions, were inoculated with seven strains; GMF14, GMM71, GEM96, GMF42, GMM34, GMM36, and GMF57. These strains were selected because in the plant test under optimal temperature conditions, they significantly increased plant biomass from that with the control non-inoculated treatment, with the exception of strain GMF57 (Table 2). Among the selected strains, strains GMF14 and GMF57 grew at 4°C (Table S3). Additionally, when inoculated into “Merlin”, these strains showed no significant difference in terms of dry weight from the positive control USDA 110.

**Fig. 6.**
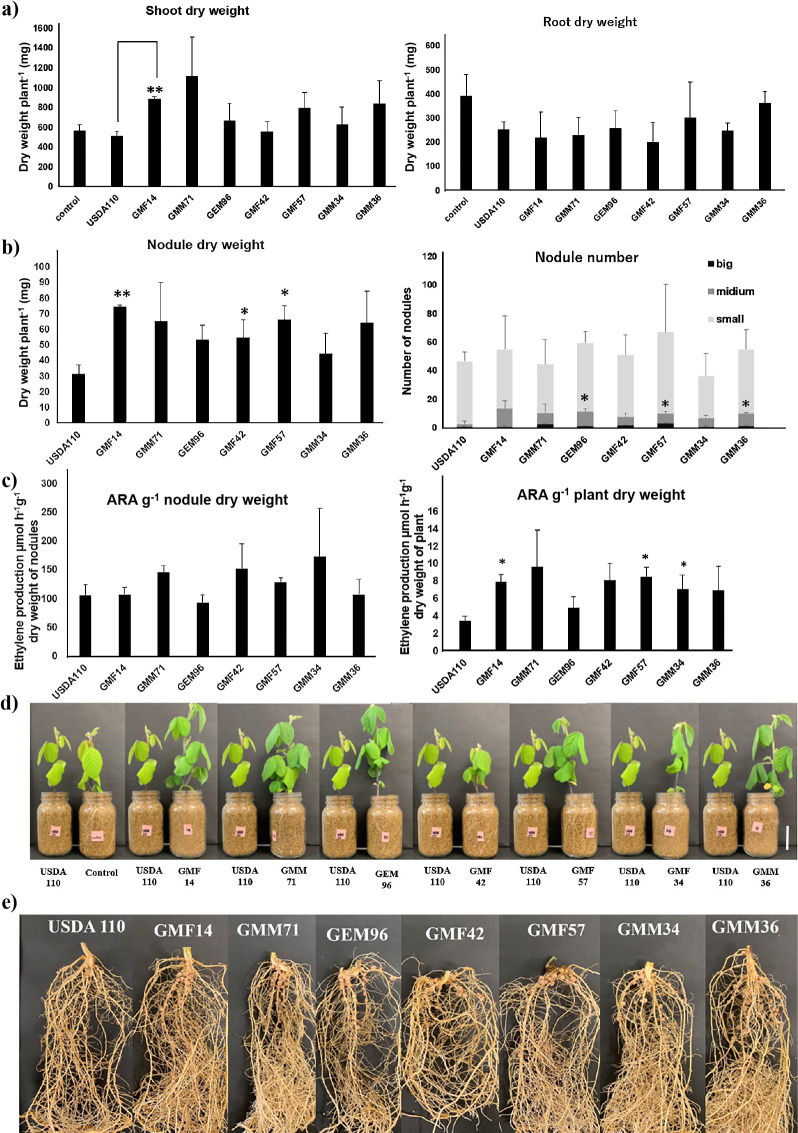
Shoot dry weight, root dry weight, root nodule dry weight, root nodule number, and acetylene reduction activity per nodule and plant of “Merlin” soybean inoculated with 7 isolates under cold conditions (the control is a non-inoculated plant). Large nodules >5 mm, medium-sized nodules 2–5 mm, and small nodules <2 mm. a) Shoot dry weight and root dry weight, b) root nodule dry weight and root nodule number, c) acetylene reduction activity nodule^–1^ dry weight and plant^–1^ dry weight, d) images of plants, and e) images of nodules. Results for each strain were compared to those for strain USDA 110 using the Student’s *t*-test; *P<0.05, **P<0.01.

Under cold conditions, the inoculation of “Merlin” with strain GMF14 significantly increased the shoot dry weight of the “Merlin” cultivar from that with USDA 110 (Fig. 6A). However, the root dry weight induced by strain GMF14 was not significantly different from that induced by USDA 110 (Fig. 6A). The nodule dry weights induced by GMF14, GMF42, and GMF57 were significantly higher than those induced by USDA 110 (Fig. 6B). Regarding total nodule numbers, no significant difference was observed between any strains, and GEM96, GMF57, and GMM36 formed more medium-sized nodules than USDA 110 (Fig. 6B); most of the nodules formed with USDA 110 were small. Regarding ARA g^–1^ of nodule dry weight, no significant difference was observed between any strains, whereas ARA (g^–1^ of plant dry weight) of plants inoculated with GMF14, GMF57, and GMM34 were significantly higher than those of USDA 110-inoculated plants (Fig. 6C). Plants inoculated with GMF14 and GMF57 appeared to have darker green leaves than those inoculated with USDA110 (Fig. 6D) as well as a significantly higher nodule dry weight and ARA g^–1^ of plant dry weight than USDA 110-inoculated plants (Fig. 6B and C).

In contrast, in the Sultana cultivar (Fig. S1), shoot dry weight, root dry weight, nodule dry weight, nodule number, ARA g^–1^ of nodule dry weight, and ARA g^–1^ of plant dry weight were not significantly higher than those of USDA 110-inoculated plants.

## Discussion

### Distribution of soybean rhizobia from the northern part of Germany

In the present study, rhizobia were isolated from eighteen soil samples collected from different areas in the northern part of Germany using two soybean cultivars, “Merlin” and “Enrei”, as trap hosts (Fig. 1). Soybean rhizobial strains were mostly isolated from fields in the Müncheberg and Fehrow areas that belong to Brandenburg State, which have soybean cultivation histories. In these areas, farmers use commercial rhizobial fertilizers from the BASF company (https://www.basf.com/global/en.html), such as HiStick^®^-soy containing *B. japonicum*. This fertilizer needs to be inoculated into soybean seeds at a rate of 4×10^9^ viable cells g^–1^ before sowing to fix nitrogen and meet yield potentials (Zimmer *et al.*, 2016). A commercial rhizobial strain was introduced into these two areas as inoculants between 2013 and 2017, after which soybean was not cultivated. Soil from the remaining 7 locations with no soybean cultivation history, such as Sachsendorf, Dedelow, Paulinenaue, Nossen, Köllitsch, Golmbach, and Schnega may have small populations of soybean rhizobia, with the exception of Köllitsch, at which several root nodules were obtained using “Enrei” as the trap host. This finding clearly show that if soybean cultivations are introduced in the above locations, the application of *Bradyrhizobium* inoculants is needed to promote symbiotic performance between soybean and *Bradyrhizobium* in order to yield maximum seed production.

### Characteristics of isolates in terms of stress tolerance

The stress tolerance characteristics of rhizobial strains are important for adaptation to the local environmental conditions of a given soil. In the present study, we attempted to identify strains with multiple stress tolerance and efficient nitrogen fixation abilities, with the major aim of selecting a rhizobial inoculant for soybean cultivation under field conditions in Germany. One of the key features that needed to be fulfilled by the screened isolates was cold tolerance because rhizobial inoculants need to adapt to the low temperatures that range between 8 and 15°C in German fields (Zimmer *et al.*, 2016). At the same time, high temperatures affect rhizobia infection and nitrogen fixation in several legume species, including soybean, peanut, and cowpea (Munevar and Wollum, 1982; Rainbird *et al.*, 1983; Kishinevsky *et al.*, 1992). Soil degradation, due to salinization, alkalization, and acidification, is one of the most serious issues affecting the fertility of soils, particularly in arid and semi-arid areas. Therefore, given the importance of the stress tolerance abilities of rhizobial strains, the isolates of the present study were screened for their growth under different stress conditions of temperature, salinity, and pH. Thirty-three isolates (15.1% of the total) trapped on the “Merlin” cultivar had the ability to grow at a low temperature, in contrast to none from “Enrei”. The cold tolerant rhizobium in German soils may exhibit stronger symbiotic ability to the German cultivar “Merlin” than to the Japanese cultivar “Enrei”; however, the mechanisms by which the survival ability at a low temperature of isolates correlates with symbiotic ability with soybean have not yet been elucidated in detail. However, to develop agricultural practices for soybean cultivation in Europe, a clearer understanding of this interaction is very important.

Furthermore, the majority of the low temperature and high NaCl tolerant strains were isolated from “Merlin” (Table S2, Fig. 2). This indicates that “Merlin” is a better trap host for isolating candidates of soybean inoculants being adapted to European soybean cultivation. This is consistent with previous findings by Artigas *et al.* (2019), which showed that domesticated soybean is a better host trap and its rhizobial strains are adapted to native abiotic stress conditions (Artigas *et al.*, 2019).

### Phylogenetic properties of isolates

To elucidate the genetic background of soybean rhizobia showing tolerance to various abiotic stresses, 77 isolates were selected and assessed for their phylogenetic properties based on a sequence analysis of their 16S rRNA, *recA*, and *atpD* genes. Therefore, we performed MLSA of the 16S rRNA, *recA*, and *atpD* genes, as shown in Fig. 3. In subgroup A, 6 isolates from the present study belonged to type strains of *B. diazoefficiens* USDA 110 and *B. japonicum* USDA 6. These type strains are currently agriculturally important rhizobia worldwide because they have superior N_2_ fixation abilities (Singleton *et al.*, 1985; Pazdernik *et al.*, 1997; Klogo *et al.*, 2015). *B. japonicum* strains are being used in the global production of commercial inoculants for soybeans, including Germany (Zimmer *et al.*, 2016). This result indicates that these *Bradyrhizobium* strains originated from commercial inoculants. In group B, the majority of strains belong to *B. lupini*, *B. yuanmingense*, and *B. elkanii*. In Germany, lupini is a common plant. Wiehe and Höflich (Wiehe and Höflich, 1995) previously reported that lupini plants were cultivated in the Müncheberg area. Lupini is nodulated by rhizobia belonging to the genus *Bradyrhizobium* (Eckhardt *et al.*, 1931; Andam and Parker, 2007). A total of 28.6% isolates were assigned to the *Rhizobium* species in the present study. Previous studies also reported the isolation of rhizobia belonging to *Rhizobium*/*Agrobacterium* from soybean plants (Abaidoo *et al.*, 2000; Chen *et al.*, 2000; Hong *et al.*, 2010; Youseif *et al.*, 2014; Alam *et al.*, 2015). The present results revealed that *Bradyrhizobium* is the predominant symbiont of soybean in the northeastern part of German soils. Furthermore, these German indigenous rhizobia are distributed in fields with soybean cultivation histories, which indicates that the natural population of rhizobia have been affected by both the cultivation of different legume crops and the application of inoculants. These results are supported by other investigations in Europe, such as Poland and Spain, and also in Africa (Madrzak *et al.*, 1995; Bourebaba *et al.*, 2016; Chibeba *et al.*, 2017).

### Genetic characterization of symbiotic genes

The phylogenetic tree, built with the partial sequences of the *nodD* and *nifH* genes, showed that all isolates, except for GMM49, possessed identical *nodD* and *nifH* genes, suggesting a common origin for these symbiotic genes. However, these symbiotic genes may be derived from a single strain that may be the commercial inoculants previously introduced in these fields. These results are consistent with previous findings between non-*Bradyrhizobium* strains and introduced *B. japonicum* inoculants in Brazil, Poland, and Africa (Martínez-Hidalgo and Hirsch, 2017). Regarding the MLSA of the 16S rRNA, *atpD*, and *recA* genes shown in Fig. 3, a large number of isolates were classified into subgroup B, which is genetically remote from subgroup A including *B. japonicum* and *B. diazoefficiens*. These results imply that the horizontal gene transfer (HGT) of symbiotic genes occurred from the commercial inoculant to indigenous soil bacteria with different genetic backgrounds of *Bradyrhizobium*. HGT may occur within several years of the introduction of commercial inoculants, suggesting rapid and frequent genome rearrangement through HGT among inoculants and soil bacteria (Barcellos *et al.*, 2007). GMM49 has the unique characteristic of possessing *R. lusitanum*-type housekeeping genes, *B. elkanii*-type *nodD* genes, and *B. diazoefficiens*-type *nifH* genes. This supports HGT potentially occurring among inoculants and soil bacteria in the fields tested, and imply the complex rearrangement of symbiotic genes in isolates recovered from the field. The HGT of nodulation and nitrogen fixation genes in a symbiosis island is well known between the same or different rhizobial genera, which supports the present results (Parker *et al.*, 2002; Barcellos *et al.*, 2007; Martínez-Hidalgo and Hirsch, 2017; Artigas *et al.*, 2019). Similar findings were reported by Artigas *et al.* (2019), who isolated *R. alamii* species with *nod* and *nifH* genes derived from *Bradyrhizobium* sp. (Artigas *et al.*, 2019). Furthermore, the complex HGT of symbiotic genes occurred in rhizobia in Brazilian Savannah soils, showing the genetic variability due to adaptation to stressful environmental conditions (Barcellos *et al.*, 2007). These findings highlight the strategies that bacteria may often use as ecological advantages, such as the acquisition of genes for symbiotic ability with an exotic host legume.

### Symbiotic performance of isolates

Soybean requires warm growing conditions with temperatures ranging between 25 to 30°C for optimal symbiotic activity. Under cold conditions, soybean shows a decrease in both nodulation and nodule function, which will directly affect soybean yield. Cold conditions negatively affect nitrogen-fixing symbiosis, mainly due to the sensitivity of the host plant and this effect may be alleviated by using cold tolerant legume and rhizobial partners. Prevost *et al.* (1987) reported that the nitrogenase activities of rhizobia isolated from cold areas were higher at low temperatures than those from warm areas. They isolated a strain from a cold environment that formed nodules under low temperatures, whereas a strain isolated from a warmer environment did not (Prevost *et al.*, 1987). Zhang *et al.* (1996) reported that two *B. japonicum* rhizobia, isolated from cool soil conditions in Canada, increased the nodule number, nodule weight, shoot nitrogen, and yield of soybean in field experiments, and also found that the implementation of rhizobia under cold conditions was affected by their geographical origin (Zhang *et al.*, 1996). Collectively, these findings imply that the optimal strategy to promote soybean nodulation and, thus, its yield in German soils is to isolate local rhizobial strains that are tolerant to German cold conditions.

Under optimal temperature conditions, plant biomass was not significantly different between the seven selected isolates and USDA 110 (Table 2). On the other hand, when strain GMF14 was inoculated into “Merlin” soybean under low temperature conditions, strain GMF14 stimulated the plant biomass, particularly shoot dry weight, and its effects were different from USDA 110 (Fig. 6). This result indicates that these bacteria isolated from cold environments stimulate soybean biomass. The cultivar “Merlin” inoculated with the *Bradyrhizobium* strain GMF14 (belonging to *Bradyrhizobium* sp. based on MLSA) increased plant growth and was tolerant of low temperate conditions. Nevertheless, when GMF 14 was inoculated into the other cultivar “Sultana”, it did not show the same results (Fig. S1). This indicates that GMF 14, which was isolated from “Merlin”, had a better symbiotic relationship with this host plant than “Sultana” under cold conditions. Based on the present study, which focused on abiotic stress, such as low temperatures, GMF14 is the most promising candidate for soybean inoculation at Brandenburg State. In the next step, we need to investigate inoculation effects using these candidate strains in field experiments under German conditions.

In conclusion, the present study is the first to attempt to identify the genetic background of indigenous soybean rhizobia isolated from northern Germany. Stress tolerance and plant test results demonstrated that there are several promising isolates for soybean inoculation in European soybean cultivation systems. Furthermore, these candidates may adapt to local environments. GMF14 induced the highest plant growth, root nodule number, and nitrogen fixation among all the isolates obtained. Further field studies are needed for the development of suitable inoculation technology for soybean cultivation in European soils, including Germany.

## Supplementary Material

Supplementary Material

## Figures and Tables

**Table 1.** Soil information from sampling sites

Soil sample no.	Site Name	Collection Date	Longitude X	Latitude Y	Soil rating index*	Clay (%)	Silt (%)	Sand (%)	Crop sequence history	Soybean cultivation	pH
1	Sachsendorf	2017/11/30	14.4690	52.5045	54	27	49	25	Oilseed rape - winter wheat	No	7.0±0.15
2	Dedelow	2017/11/9	13.8016	53.3696	45	10	32	58	Winter wheat - maize - maize - winter wheat - oilseed rape - winter barley - winter wheat	No	6.9±0.05
3	Paulinenaue	2017/11/22	12.6689	52.6474	45	9	20	72	Oilseed rape - winter barley - winter wheat	No	6.8±0.02
4	Nossen	2017/11/17	13.2693	51.0567	63	16	81	4	Pea - oat - clover - winter wheat - potato	No	6.5±0.06
5	Köllitsch	2017/11/17	13.0942	51.5136	64	14	34	52	Pea - winter wheat - alfalfa - alfalfa - alfalfa - maize - winter wheat	No	6.6±0.02
6	Golmbach	2017/11/28	9.5381	51.9029	75	15	77	9	Maize - winter wheat - winter barley - maize	No	6.8±0.02
7	Schnega	2017/11/28	10.8330	52.9016	25	3	5	93	Flower mixtures - crops	No	6.0±0.18
8	Müncheberg	2017/11/30	14.1274	52.5156	29	4	10	86	Grass - grass - grass	No	6.4±0.07
9	Müncheberg	2017/11/30	14.1287	52.5205	30	4	10	86	Maize - alfalfa - winter rye - winter rye - alfalfa - alfalfa	No	6.7±0.07
10	Müncheberg	2017/11/30	14.1227	52.5185	30	4	10	86	Oat - grass/clover - oat - **soybean** - maize - alfalfa	2014	6.8±0.06
11	Müncheberg	2017/11/30	14.1281	52.5214	30	4	10	86	Alfalfa - oat - **soybean** - maize - alfalfa - winter rye	2015	6.5±0.06
12	Müncheberg	2017/11/30	14.1326	52.5227	30	4	10	86	Oat - **soybean** - winter barley - winter wheat	2016	6.1±0.03
13	Müncheberg	2017/11/30	14.1340	52.5223	30	4	10	86	**Soybean** - winter wheat - oilseed rape - winter rye	2017	6.3±0.04
14	Fehrow	2017/11/29	14.2504	51.8359	27	5	15	80	Winter rye - **soybean**	2013	6.6±0.02
15	Fehrow	2017/11/29	14.2759	51.8526	27	5	15	80	Maize - grass/clover - winter wheat - **soybean** - maize -grass/clover	2014	6.3±0.02
16	Fehrow	2017/11/29	14.2720	51.8293	43	5	15	80	Grass/clover - winter wheat - **soybean** - maize - grass/clover - grass/clover	2015	6.3±0.02
17	Fehrow	2017/11/29	14.2782	51.8543	31	5	15	80	Winter wheat - **soybean** - winter wheat - grass/clover - winter wheat - lupini	2016	6.4±0.02
18	Fehrow	2017/11/29	14.2412	51.8362	36	5	15	80	winter wheat - grass/clover - winter wheat - **soybean**	2017	6.3±0.01

* The German soil rating index describes the yield potential of soil ranging from very poor soil with a rating of 10 to very good soil with a rating of 100. Data on the soil rating index, soil texture, and crop sequence history were measured and monitored by research stations at each location.

**Table 2.** Summary of results of the phylogenetic analysis and symbiotic performance of 22 *Bradyrhizobium* from German soils and *B. diazoefficiens* USDA 110

Soil sample no.	Sampling sites	Isolates	Phylogeny based on MLSA	Host plants	Biomass^a^ (Merlin)	Biomass^a^ (Enrei)	NN^c^ (Merlin)	NN^c^ (Enrei)	ARA^b^ (Merlin)	ARA^b^ (Enrei)
Control	Non-inoculation	Control	—	—	523.6±103.4	1529.4±185.0	0.0	0.0	0.0	0.0
—	—	USDA 110	*B. diazoefficiens*	—	1195.0±107*	1727.7±35.8	38.7±13.6	22.7±4.5	89.9±31.3	52.1±11.7
8	Müncheberg	GEM94	*Bradyrhizobium* sp.	‘Enrei’	545.7±210.0	1827±306.1	18.2±9.7	35.7±13.1	42.3±10.2	16.7±7.6
9	Müncheberg	GEM96	*Bradyrhizobium* sp.	‘Enrei’	884.0±128.8*	1909.8±517.7	28.6±14.7	40.7±9.2	77.5±13.7	50.6±10.6
9	Müncheberg	GEM95	*Bradyrhizobium* sp.	‘Enrei’	1014.0±253.0	1754.4±301.9	23.3±8.1	45±21.3	39.2±7.0	34.3±8.1
10	Müncheberg	GMM65	*Bradyrhizobium* sp.	‘Merlin’	904.0±149.9	1789.3±396.7	38.3±8.9	31.3±10.7	44.5±28.4	21.3±3.8
11	Müncheberg	GMM71	*Bradyrhizobium* sp.	‘Merlin’	921.7±57.5*	2191.8±53.8**	37.3±10.7	30.0±2.4	42.1±5.2	47.9±23.6
11	Müncheberg	GEM100	*Bradyrhizobium* sp.	‘Enrei’	951.3±527.0	2173.3±116.6	30.3±15.5	37±3.2	87.7±37.8	74.1±40.3
12	Müncheberg	GMM29	*Bradyrhizobium* sp.	‘Merlin’	665.4±95.4	1609.2±151.3	33.3±12.9	33.3±14.0	44.1±6.5	65.9±40.6
13	Müncheberg	GMM36	*Bradyrhizobium* sp.	‘Merlin’	1010.8±190.6*	1939.4±205.0	26.0±17.9	35±8.5	38.3±19.6	30.0±4.7
13	Müncheberg	GMM34	*B. japonicum*	‘Merlin’	1010.8±190.6*	1762.3±549.1	35.3±3.8	34.7±13.4	81.7±12.1	23.6±16.8
13	Müncheberg	GEM108	*Bradyrhizobium* sp.	‘Enrei’	977.6±196.0*	1536.1±171.7	31.7±7.6	48.7±16.2	66.9±11.1	11.1±7.2
5	Köllitsch	GEK77	*Bradyrhizobium* sp.	‘Enrei’	757.7±381.0	1830.0±573.5	23.3±14.3	24.3±10.7	64.0±5.4	28.1±19.8
14	Fehrow	GMF57	*Bradyrhizobium* sp.	‘Merlin’	592.5±53.7	1829.6±215.5	39.0±9.9	43.3±19.7	42.0±17.9	94.8±7.3
15	Fehrow	GEF111	*Bradyrhizobium* sp.	‘Enrei’	888.7±169.0	1827.6±392.0	21.0±5.0	37±10.3	101.4±35.5	19.2±1.4
15	Fehrow	GMM44	*Bradyrhizobium* sp.	‘Merlin’	781.9±312.5	1942.1±216.4	26.0±20.7	46±7.16	55.3±26.5	29.8±1.3
15	Fehrow	GMF42	*Bradyrhizobium* sp.	‘Merlin’	923.7±198.0*	1710.7±119.4	61.3±29.1	28.3±4.4	60.5±38.8	81.3±48.6
16	Fehrow	GMF19	*Bradyrhizobium* sp.	‘Merlin’	691.0±76.9	1992.1±376.2	54.3±15.9	42.3±12.2	43.2±16.9	51.6±22
16	Fehrow	GMF24	*Bradyrhizobium* sp.	‘Merlin’	571.8±197.8	1474.9±322.1	40.7±9.9	27±10.2	140.6±11.6	19.3±6.8
16	Fehrow	GEF112	*Bradyrhizobium* sp.	‘Enrei’	853.3±311.0	1858.9±501.2	27.0±5.6	46.7±16.5	90.4±16.1	31.1±23.9
17	Fehrow	GEF115	*Bradyrhizobium* sp.	‘Enrei’	967.3±276.0	1844.1±425.2	23.3±15.4	30.7±11.9	38.3±12.6	31.1±23.9
17	Fehrow	GMF10	*Bradyrhizobium* sp.	‘Merlin’	682.8±323.8	1390.6±395.5	38.0±18.5	49.3±11.3	56.4±16.1	24.6±1.6
18	Fehrow	GEF118	*Bradyrhizobium* sp.	‘Enrei’	740.0±305.0	1495.5±264.2	26.0±16.4	27.7±6.7	101.5±29.1	17.6±3.6
18	Fehrow	GMF14	*Bradyrhizobium* sp.	‘Merlin’	892.8±183.2*	2113.2±140.2*	61.3±26.0	33±3.9	61.7±14.3	54.6±13.5
8	Müncheberg	GMM49	*R. lusitanum*	‘Merlin’	428.9±149.5	1547.8±252.3	35.7±13.2	0.0	119.8±28.6	0.0

^a^ Biomass of the plant 4‍ ‍weeks after the inoculation (mean±standard deviation; *n*=4). Control plants (non-inoculated) had no nodules. The plant test was performed with *G. max* cultivar Merlin and Enrei (mg plant^–1^). Results for each strain were compared to a non-inoculated control using the Student’s *t*-test; *P<0.05, **P<0.01. ^b^ Acetylene reduction assay (ARA). Values represent activity expressed as μmol C_2_H_4_^–1^ h^–1^ g^–1^ dry weight of nodules. ^c^ Nodule number (NN) (mg plant^–1^).
