# Supplementary material for: Characterization of Rhizobia for the Improvement of Soybean Cultivation at Cold Conditions in Central Europe
Source: Microbes Environ. 2020 Jan 30;35(1):ME19124. doi: 10.1264/jsme2.ME19124 (PMC7104276; doi:10.1264/jsme2.ME19124)
Supplement: Supplementary file 1 — Supplementary Material [file 35_19124_s1.pdf]

Table S1. Primers used for PCR amplification of 16S rRNA, *recA*, *atpD*, *nodD* and *nifH* genes.

Primer	sequence（ 5´- 3´）	Target gene	PCR conditions	Reference
16AF	AACTGAAGAGTTTGATCMTGGCTCAG	16S rRNA	3min 95℃, 30×(1min 94℃, 45s	Frank *et al*. (2008)
1492R	TACGGYTACCTTGTTACGACTT	16S rRNA	54℃, 2min 72℃), 7min 72℃	
*recA*F	CGKCTSGTAGAGGAYAAATCGGTGGA	*recA*	5min 95℃, 35×(45s 94℃, 1min	Gaunt *et al*. (2001)
*recA*R	CGRATCTGGTTGATGAAGATCACCAT	*recA*	40℃, 90s 74℃), 5min 74℃	
*atpD*F	SCTGGGSCGYATCMTGAACGT	*atpD*	3min 95℃, 35×(45s 94℃, 1min	Gaunt *et al*. (2001)
*atpD*R	GCCGACACTTCCGAACCNGCCTG	*atpD*	55℃, 90s 72℃), 5min 72℃	
nodDF	TTATGGCCCGACATCCGTTCGAC	*nodD*	5min 95℃, 30×(1min 94℃, 2min	Risal *et al*. (2010)
*nodD*R	CCAACTATGAGAGGCGGGATCAT	*nodD*	55℃, 3min 72℃), 5min 72℃	
*nifH*F	TACGGNAARGGSGGNATCGGCAA	*nifH*	2min 95℃, 35×(30s 95℃, 30s	Laguerre *et al*. (2001)
*nifH*R	AGCATGTCYTCSAGYTCNTCCA	*nifH*	57℃, 1min 72℃), 5min 72℃	

Frank, J. A., Reich, C. I., Sharma, S., Weisbaum, J. S., Wilson, B. A., and Olsen, G. J. (2008) Critical evaluation of two primers commonly used for amplification of bacterial 16S rRNA genes. *Appl Environ Microbiol* **74**: 2461– 2470.

Gaunt, M.W., Turner, S.L., Rigottier-Gois, L., Lloyd-Macgilp, S.A., and Young, J.P. (2001) Phylogenies of *atpD* and *recA* support the small subunit rRNA-based classification of rhizobia. *Int J Syst Evol Microbiol* **51**: 2037–2048.

Risal, C.P., Yokoyama, T., Ohkama-Ohtsu, N., Djedidi, S., and Sekimoto, H. (2010) Genetic diversity of native soybean *Bradyrhizobia* from different topographical regions along the southern slopes of the Himalayan Mountains in Nepal. *Syst Appl Microbiol* **33**: 416–425.

Laguerre, G., Nour, S.M., Macheret, V., Sanjuan, J., Drouin, P., and Amargar, N. (2001) Classification of rhizobia based on *nodC* and *nifH* gene analysis reveals a close phylogenetic relationship among *Phaseolus vulgaris* symbionts. *Microbiol* **147**: 981–993.

Table S2. The number of nodules isolated from two soybeans cultivars in different areas, as well as their size, stress tolerance ability and, the number of strains used for phylogenetic analysis.

Host plant	Soil sample no.	No. of isolations	No. of big size nodules	N0. of middle size nodules	No. of small size nodules	No. of strians tolerant of 4 ℃	No. of strians tolerant of 1%-4% NaCl	No. of strians tolerant of pH 4-10	No. of fast growing strians	No. of slow growing strains	No. of strians used for plyogenetic analysis
G. max 'Merlin'	1	0	0	0	0	0	0	0	0	0	0
	2	0	0	0	0	0	0	0	0	0	0
	3	0	0	0	0	0	0	0	0	0	0
	4	0	0	0	0	0	0	0	0	0	0
	5	0	0	0	0	0	0	0	0	0	0
	6	0	0	0	0	0	0	0	0	0	0
	7	0	0	0	0	0	0	0	0	0	0
	8	2	0	0	2	0	0	2	0	2	1
	9	0	0	0	0	0	0	0	0	0	0
	10	31	5	18	8	2	5	30	5	26	4
	11	33	2	16	15	2	3	32	5	28	5
	12	29	1	22	6	1	1	29	10	19	5
	13	19	9	8	2	1	2	19	1	18	5
	14	28	6	18	4	6	6	28	4	24	2
	15	24	1	24	0	4	3	24	1	23	5
	16	25	8	15	2	9	6	25	11	14	8
	17	38	0	18	20	11	10	38	15	23	8
	18	35	0	22	13	10	12	34	15	20	1
G. max 'Enrei'	1	0	0	0	0	0	0	0	0	0	0
	2	0	0	0	0	0	0	0	0	0	0
	3	0	0	0	0	0	0	0	0	0	0
	4	0	0	0	0	0	0	0	0	0	0
	5	3	1	0	2	0	0	3	0	3	3
	6	0	0	0	0	0	0	0	0	0	0
	7	0	0	0	0	0	0	0	0	0	0
	8	3	0	0	3	0	0	3	0	3	3
	9	2	0	0	2	0	0	2	0	2	2
	10	20	4	10	6	0	0	20	0	20	2
	11	21	7	10	4	0	0	20	0	21	3
	12	20	2	11	7	0	0	20	0	20	3
	13	20	6	12	2	0	0	20	0	20	2
	14	21	5	10	6	0	0	20	0	21	3
	15	20	7	6	7	0	2	20	0	20	3
	16	20	6	7	7	0	0	20	0	20	3
	17	20	5	8	7	0	0	19	0	20	3
	18	20	7	8	5	0	2	19	0	20	3

* Size of nodules : big size nodules >5 mm, middle sieze of nodules 2-5 mm and small size of nodules < 2 mm.

* Growth rate: fast growing(1-3 days), slow growing (4-7 days).

Table S3. Summary of phylogenetic analysis of the MLSA and stress tolerance of isolates to NaCl, pH and

Strain	Host plant	Samping sites	Soil no.	Last soyben history	MLSA	Fast growth	Slow growth	Temparature range (°C)	NaCl(%)	pH
**GMF1**	'Merlin'	Fehrow	17	2016	*R. pusense*		+	4-37	0%	4.5-10
**GMF2**	'Merlin'	Fehrow	17	2016	*R. lusitanum*	+		4-37	0%	4.5-10
**GMF3**	'Merlin'	Fehrow	17	2016	*R. lusitanum*	+		4-37	0%	4.5-10
**GMF4**	'Merlin'	Fehrow	17	2016	*R. pusense*	+		4-37	4%	4.5-10
**GMF6**	'Merlin'	Fehrow	17	2016	*R. pusense*	+		4-37	2%	4.5-10
**GMF7**	'Merlin'	Fehrow	17	2016	*R. pisi / R. alamii*	+		4-37	4%	4.5-10
**GMF8**	'Merlin'	Fehrow	17	2016	*R. pusense*	+		4-37	4%	4.5-10
**GMF9**	'Merlin'	Fehrow	17	2016	*R. pisi /R. alamii*	+		15-37	4%	4.5-10
**GMF10**	'Merlin'	Fehrow	18	2017	*Bradyrhizobium* sp.		+	15-37	2%	4.5-10
**GMF14**	'Merlin'	Fehrow	16	2015	*Bradyrhizobium* sp.		+	4-37	4%	4.5-10
**GMF18**	'Merlin'	Fehrow	16	2015	*R. pisi / R. alamii*	+		4-37	0%	4.5-10
**GMF19**	'Merlin'	Fehrow	16	2015	*Bradyrhizobium* sp.		+	15-37	0%	4.5-10
**GMF23**	'Merlin'	Fehrow	16	2015	*R. lusitanum*	+		4-37	0%	4.5-10
**GMF24**	'Merlin'	Fehrow	16	2015	*Bradyrhizobium* sp.		+	15-37	0%	4.5-10
**GMF25**	'Merlin'	Fehrow	16	2015	*R. lusitanum*	+		4-37	0%	4.5-10
**GMF26**	'Merlin'	Fehrow	16	2015	*R. lusitanum*	+		4-37	2%	4.5-10
**GMF27**	'Merlin'	Fehrow	16	2015	*R. lusitanum*	+		15-37	3%	4.5-10
**GMM28**	'Merlin'	Müncheberg	12	2016	*Bradyrhizobium* sp.		+	15-37	0%	4.5-10
**GMM29**	'Merlin'	Müncheberg	12	2016	*Bradyrhizobium* sp.		+	15-37	0%	4.5-10
**GMM30**	'Merlin'	Müncheberg	12	2016	*Bradyrhizobium* sp.		+	15-37	0%	4.5-10
**GMM31**	'Merlin'	Müncheberg	12	2016	*B. japonicum*		+	15-37	1%	4.5-10
**GMM32**	'Merlin'	Müncheberg	12	2016	*B. japonicum*		+	15-37	0%	4.5-10
**GMM34**	'Merlin'	Müncheberg	13	2017	*B. japonicum*		+	15-37	0%	4.5-10
**GMM35**	'Merlin'	Müncheberg	13	2017	*Bradyrhizobium* sp.		+	15-37	0%	4.5-10
**GMM36**	'Merlin'	Müncheberg	13	2017	*Bradyrhizobium* sp.		+	15-37	0%	4.5-10
**GMM37**	'Merlin'	Müncheberg	13	2017	*Bradyrhizobium* sp.		+	15-37	0%	4.5-10
**GMM40**	'Merlin'	Müncheberg	13	2017	*Bradyrhizobium* sp.		+	15-37	0%	4.5-10
**GMF41**	'Merlin'	Fehrow	15	2014	*R. pusense*	+		4-37	4%	4.5-10
**GMF42**	'Merlin'	Fehrow	15	2014	*Bradyrhizobium* sp.		+	15-37	0%	4.5-10
**GMF43**	'Merlin'	Fehrow	15	2014	*R. pusense*		+	15-37	0%	4.5-10
**GMF44**	'Merlin'	Fehrow	15	2014	*Bradyrhizobium* sp.		+	15-37	0%	4.5-10
**GMF46**	'Merlin'	Fehrow	15	2014	*Bradyrhizobium* sp.		+	15-37	0%	4.5-10
**GMM49**	'Merlin'	Müncheberg	8	no	*R. lusitanum*		+	15-37	0%	4.5-10

**GMF55**	'Merlin'	Fehrow	14	2013	*R. pisi / R. alamii*	+		4-37	4%	4.5-10
**GMF57**	'Merlin'	Fehrow	14	2013	*Bradyrhizobium* sp.		+	4-37	3%	4.5-10
**GMM59**	'Merlin'	Müncheberg	10	2014	*R. lusitanum*	+		4-37	1%	4.5-10
**GMM60**	'Merlin'	Müncheberg	10	2014	*R. lusitanum*	+		15-37	0%	4.5-10
**GMM64**	'Merlin'	Müncheberg	10	2014	*R. lusitanum*	+		4-37	2%	4.5-10
**GMM65**	'Merlin'	Müncheberg	10	2014	*Bradyrhizobium* sp.		+	15-37	0%	4.5-10
**GMM67**	'Merlin'	Müncheberg	11	2015	*Bradyrhizobium* sp.		+	15-37	0%	4.5-10
**GMM68**	'Merlin'	Müncheberg	11	2015	*R. lusitanum*	+		15-37	4%	4.5-10
**GMM69**	'Merlin'	Müncheberg	11	2015	*R. lusitanum*	+		4-37	1%	4.5-10
**GMM70**	'Merlin'	Müncheberg	11	2015	*Bradyrhizobium* sp.		+	15-37	0%	4.5-10
**GMM71**	'Merlin'	Müncheberg	11	2015	*Bradyrhizobium* sp.		+	15-37	0%	4.5-10
**GEK77**	'Enrei'	Köllitsch	5	no	*Bradyrhizobium* sp.		+	15-37	0%	4.5-10
**GEK79**	'Enrei'	Köllitsch	5	no	*B. japonicum*		+	15-37	0%	4.5-10
**GEK80**	'Enrei'	Köllitsch	5	no	*Bradyrhizobium* sp.		+	15-37	0%	4.5-10
**GEF82**	'Enrei'	Fehrow	14	2013	*Bradyrhizobium* sp.		+	15-37	0%	4.5-10
**GEF83**	'Enrei'	Fehrow	14	2013	*Bradyrhizobium* sp.		+	15-37	0%	4.5-10
**GEF84**	'Enrei'	Fehrow	14	2013	*Bradyrhizobium* sp.		+	15-37	0%	4.5-10
**GEM87**	'Enrei'	Müncheberg	8	no	*Bradyrhizobium* sp.		+	15-37	0%	4.5-10
**GEM93**	'Enrei'	Müncheberg	8	no	*Bradyrhizobium* sp.		+	15-37	0%	4.5-10
**GEM94**	'Enrei'	Müncheberg	8	no	*Bradyrhizobium* sp.		+	15-37	0%	4.5-10
**GEM95**	'Enrei'	Müncheberg	9	no	*Bradyrhizobium* sp.		+	15-37	0%	4.5-10
**GEM96**	'Enrei'	Müncheberg	9	no	*Bradyrhizobium* sp.		+	15-37	0%	4.5-10
**GEM97**	'Enrei'	Müncheberg	10	2014	*B. japonicum*		+	15-37	0%	4.5-10
**GEM99**	'Enrei'	Müncheberg	10	2014	*Bradyrhizobium* sp.		+	15-37	0%	4.5-10
**GEM100**	'Enrei'	Müncheberg	11	2015	*Bradyrhizobium* sp.		+	15-37	0%	4.5-10
**GEM101**	'Enrei'	Müncheberg	11	2015	*Bradyrhizobium* sp.		+	15-37	0%	4.5-10
**GEM102**	'Enrei'	Müncheberg	11	2015	*B. japonicum*		+	15-37	0%	4.5-10
**GEM103**	'Enrei'	Müncheberg	12	2016	*Bradyrhizobium* sp.		+	15-37	0%	4.5-10
**GEM104**	'Enrei'	Müncheberg	12	2016	*Bradyrhizobium* sp.		+	15-37	0%	4.5-10
**GEM105**	'Enrei'	Müncheberg	12	2016	*Bradyrhizobium* sp.		+	15-37	0%	4.5-10
**GEM107**	'Enrei'	Müncheberg	13	2017	*Bradyrhizobium* sp.		+	15-37	0%	4.5-10
**GEM108**	'Enrei'	Müncheberg	13	2017	*Bradyrhizobium* sp.		+	15-37	0%	4.5-10
**GEF109**	'Enrei'	Fehrow	15	2014	*Bradyrhizobium* sp.		+	15-37	0%	4.5-10
**GEF110**	'Enrei'	Fehrow	15	2014	*Bradyrhizobium* sp.		+	15-37	0%	4.5-10
**GEF111**	'Enrei'	Fehrow	15	2014	*Bradyrhizobium* sp.		+	15-37	0%	4.5-10

**GEF112**	'Enrei'	Fehrow	16	2015	*Bradyrhizobium* sp.		+	15-37	0%	4.5-10
**GEF113**	'Enrei'	Fehrow	16	2015	*Bradyrhizobium* sp.		+	15-37	0%	4.5-10
**GEF114**	'Enrei'	Fehrow	16	2015	*Bradyrhizobium* sp.		+	15-37	0%	4.5-10
**GEF115**	'Enrei'	Fehrow	17	2016	*Bradyrhizobium* sp.		+	15-37	0%	4.5-10
**GEF116**	'Enrei'	Fehrow	17	2016	*Bradyrhizobium* sp.		+	15-37	0%	4.5-10
**GEF117**	'Enrei'	Fehrow	17	2016	*Bradyrhizobium* sp.		+	15-37	0%	4.5-10
**GEF118**	'Enrei'	Fehrow	18	2017	*Bradyrhizobium* sp.		+	15-37	0%	4.5-10
**GEF119**	'Enrei'	Fehrow	18	2017	*Bradyrhizobium* sp.		+	15-37	0%	4.5-10
**GEF120**	'Enrei'	Fehrow	18	2017	*Bradyrhizobium* sp.		+	15-37	0%	4.5-10

Fig. S1 Yuan et al.

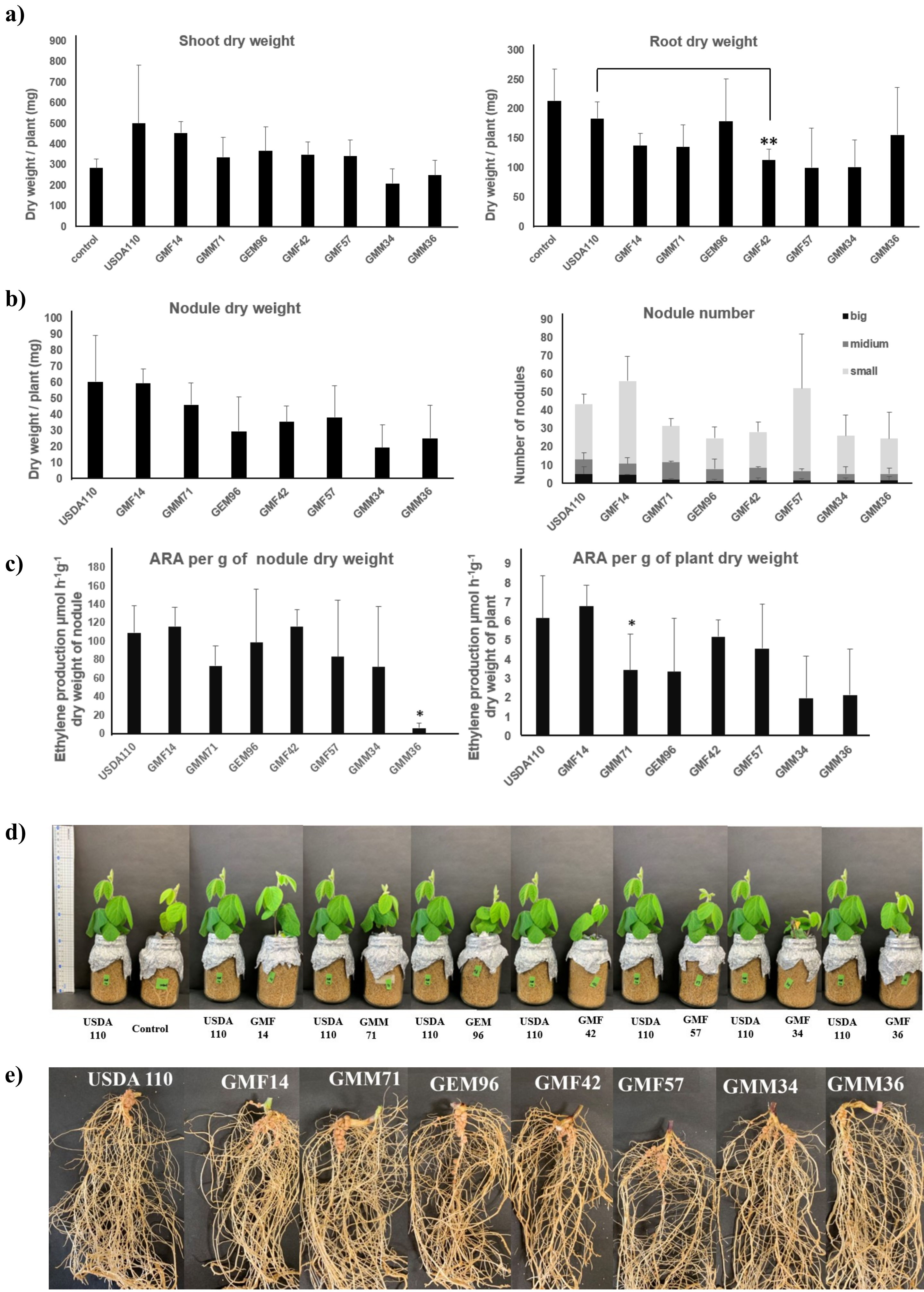

Fig. S1. Shoot dry weight, root dry weight, root nodules dry weight, root nodule numbers and acetylene reduction activity per nodules and plant of 'Sultana' soybean inoculated with the 7 isolates in cold condition (control is non-inoculated plant). Big size of nodules >5mm, medium size of nodules 2-5mm and small size of nodules <2mm. a) is Shoot dry weight and root dry weight, b) root nodules dry weight and root nodule numbers, c) is acetylene reduction activity per nodule dry weight and per plant dry weight, d) is pictures of plant, e) is pictures of nodules. Results for each strain were compared to strain USDA 110 using the student's t-test; *P<0.05, **P<0.01.
